# Statistical methods for clinical trials interrupted by the severe acute respiratory syndrome-coronavirus-2 (SARS-CoV-2) pandemic: A review

**DOI:** 10.1177/09622802241288350

**Published:** 2024-10-30

**Authors:** Joydeep Basu, Nicholas Parsons, Tim Friede, Nigel Stallard

**Affiliations:** 1 Clinical Trials Unit, Warwick Medical School, 2707 University of Warwick, Coventry, UK; 2 Department of Medical Statistics, University Medical Center Göttingen, Germany; 3 DZHK (German Center for Cardiovascular Research), partner site Göttingen, Germany

**Keywords:** COVID-19, longitudinal outcome, monotone missingness, imputation, modelling, covariate adjustment, simulation, estimands

## Abstract

Cancellation or delay of non-essential medical interventions, limitation of face-to-face assessments or outpatient attendance due to lockdown restrictions, illness or fear of hospital or healthcare centre visits, and halting of research to allow diversion of healthcare resources to focus on the pandemic led to the interruption of many clinical trials during the severe acute respiratory syndrome-coronavirus-2 (SARS-CoV-2) pandemic. Appropriate analysis approaches are now required for these interrupted trials. In trials with long follow-up and longitudinal outcomes, data may be available on early outcomes for many patients for whom final, primary outcome data were not observed. A natural question is then how these early data can best be used in the trial analysis. Although recommendations are available from regulators, funders, and methodologists, there is a lack of a review of recent work addressing this problem. This article reports a review of recent methods that can be used in the setting of the analysis of interrupted clinical trials with longitudinal outcomes with monotone missingness. A search for methodological papers published during the period 2020–2023 identified 43 relevant publications. We categorised these articles under the four broad themes of missing value imputation, modelling and covariate adjustment, simulation and estimands. Although motivated by the interruption due to SARS-CoV-2 and the resulting disease, the papers reviewed and methods discussed are also relevant to clinical trials interrupted for other reasons, with follow-up discontinued.

## 1. Introduction

The severe acute respiratory syndrome-coronavirus-2 (SARS-CoV-2) pandemic had an unprecedented impact on many aspects of human life across the globe, with, according to figures from the World Health Organisation (https://covid19.who.int/), more than 776 million infected and around 7.1 million deaths by the end of May 2024. One of the affected areas is that of clinical research. Cancellation of non-essential medical procedures, restriction of face-to-face assessments or outpatient non-attendance due to lockdown restrictions, illness or fear of hospital or healthcare centre visits, and halting of research to allow diversion of healthcare resources to focus on the pandemic (see, e.g. in the UK setting (https://www.community.healthcare.mic.nihr.ac.uk/news/the-nihrs-response-to-covid-19)), all resulted in interruption of ongoing clinical trials in the process of recruitment and/or data collection. Other trials have faced protocol changes or cycles of stopping and restarting.

A number of specific examples of trials that were interrupted by the pandemic are given by Kunz et al.^1^ These include the two-stage ATLANTE-1 trial (NCT02654587) comparing two therapies for lung cancer patients. The first stage was planned for a small sample size, which was completed, and the second stage was interrupted due to the pandemic because lung cancer patients are highly vulnerable to SARS-CoV-2 infection.

The challenges of SARS-CoV-2 and COVID-19 have resulted in many trials being stopped early with incomplete datasets. We will refer to such trials as being *interrupted*. Appropriate analysis methods are therefore required to ensure that as much information can be gained as possible from these trials.

One particular common setting on which we will focus is that of trials with long-term follow-up with outcomes measured at a number of time points throughout the follow-up period after treatments are administered. In such a trial, an interruption will often lead to data that are missing in a monotone fashion; if a patient is missing at any given follow-up stage, they are also missing at all follow-up stages. The number of observations over the sequence of time points is thus reduced sequentially due to the interruption of the study. There may also be some missing data from earlier time points due to other reasons. However, interruption may also cause non-monotone intermittent missingness, depending on the nature of interruption. For instance, an interruption caused by the SARS-CoV-2 pandemic that persisted throughout the follow-up period of a longitudinal trial might cause monotone missingness, but if an interruption lasts for a shorter period with data collection subsequently resumed, missingness could be non-monotone and intermittent.

There are guidelines on interrupted trials from regulators,^2–5^ funders (www.nihr.ac.uk/documents/restart-framework/24886) and methodologists^6^ that have described how future actions, such as stopping, pausing or restarting of a trial may be considered. There are also a number of papers on different approaches to the analysis of incomplete datasets, or datasets with monotone missingness. The purpose of this paper is to provide a review of methods for the analysis of interrupted trials, particularly those with monotone missing data. Our aim is to summarise the literature in this area, categorising the papers identified in the review into broad themes with related methods and to critically assess how they can be used in this setting. The work will provide a resource for those faced with the challenges associated with the analysis of interrupted trials.

The paper is organised as follows: Section 2 describes the methodology used to conduct the literature search. Section 3 then gives a brief summary of the number of papers identified by the review, with these papers classified into themes, with the papers within each theme briefly described. The paper concludes with a summary of findings and suggestions for future research work in Section 4.

Although motivated by trials interrupted by the SARS-CoV-2 pandemic, the work is also relevant to any trial faced with similar interruptions in which follow-up is discontinued, as discussed in Section 4.

## 2. Review methodology

In order to get a snapshot of state-of-the-art methodology for the area, and in particular to identify papers motivated by interruptions due to the SARS-CoV-2 pandemic, we used Scopus (www.scopus.com) to conduct a search for relevant articles on statistical methods for clinical trials with interruptions, dropouts or missing data published in the period 2020–2023. In order to select papers on applied statistical methods, we restricted the search to articles published or available online ahead of print in any of the 21 journals that we felt were those that published articles on the methodological aspects of clinical trials. This search was conducted on 19 April 2024. The exact details of the search terms used, including the full list of journals from which papers were drawn, are given in Appendix 1.

Papers identified using the search strategy defined above were then screened on the basis of their title and abstract to exclude those that fell outside of the focus of this review, that is, they did not present methodological work on interrupted trials dealing with missingness of longitudinal outcomes. Titles and abstracts were reviewed independently by JB and NS. For any papers where there was initial disagreement or uncertainty over whether or not they should be included in the review, consensus was reached through discussion. This led to an agreed final list of articles to be included in the review.

The full texts of all articles identified for inclusion in the review were then read and briefly summarised. A small number of key themes were then found based on the papers identified and papers were allocated to these themes. Papers that could be placed into more than one theme were assigned to those that were considered to be the closest fit. References cited in all papers included in the review were also considered to identify key older methodological work underpinning the approach used within each theme.

## 3. Review results

### 3.1. Review summary

A search conducted on 19 April 2024, using the search string given in Appendix 1 identified a total of 308 articles. Focusing on our inclusion criteria, on the basis of the title and abstract of the articles as described above, 265 articles were excluded. This led to a final list of 43 articles. These 43 papers are marked with an asterisk in the reference list at the end of the paper and when cited in the sections below.

We reviewed those papers extensively and categorised them based on themes of work, identifying four major themes of *Missing value imputation methods*, *Modelling and covariate adjustment approaches* (that do not use imputed missing values), *Simulation methods* and *Estimand methods*.

**Figure 1.**
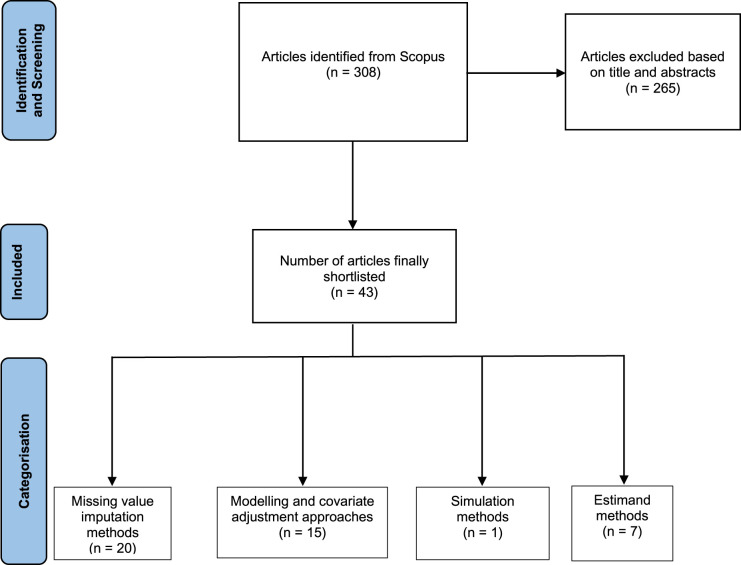
PRISMA diagram showing selection and categorisation of articles in the review.

Figure 1 presents a PRISMA diagram^7^ illustrating the selection and categorisation of papers.

The papers included in the review in each of these four themes are described in the next section together with a brief overview of each area including key references cited by the papers identified.

### 3.2. Methodological approaches identified by the review

Table 1 shows the articles published during 2020–2023 primarily included in our review. It also gives the categorisation and a brief description of the main working area.

**Table 1.** Table showing brief description of the articles included in our primary review.

Ref no.	Paper	Focus of working area
**Theme: Missing value imputation methods**		
12	Fan et al. (2020) *Stat Biopharm Res*	Mean rank imputation for non-normal outcomes
17	Lu (2021)* Stat Biopharm Res*	RTB (RBI) procedure
18	Bartlett (2023) *Stat Biopharm Res*	RBI procedure
19	Jin and Fang (2022) *J Biopharm Stat*	RBI in binary outcomes
20	Diao et al. (2022) *Stat Biopharm Res*	RBI for informative dropout
21	Wolbers et al. (2022) *Pharm Stat*	RBI for normal outcomes
24	Garcia-Hernandez et al. (2023) *Stat Biopharm Res*	RBI, JR and tipping-point analysis
25	Rabe and Bell (2020) *Stat Biopharm Res*	Different MI procedures via type I error rate and power
27	Wang et al. (2023) *Pharm Stat*	Different MI procedures for continuous outcomes under MNAR assumptions
28	Liu et al. (2023) *Stat Methods Med Res*	Imputation procedure which gives consistent variance estimator
29	Jin (2022) *Contemp Clin Trials*	RTB procedure for both MAR and MNAR simultaneously
30	Qu and Dai (2022) *Pharm Stat*	RTB procedure implemented for R and SAS
31	Zhang et al. (2022) *Stat Biopharm Res*	RTB under ’wash-out’ assumptions
32	Jin and Liu (2023) *J Biopharm Stat*	RTB with daily PRO
33	Jin et al. (2020) *Stat Methods Med Res*	Modelling to overcome bias in cycle average
34	Yang et al. (2023) *Biometrics*	MI in censored survival data
35	Jin et al. (2021) *Contemp Clin Trials*	Likelihood-based approach to differentiate between MAR and MNAR
36	Chen et al. (2021) *Contemp Clin Trials*	Hybrid imputation procedure to assess the impact of MAR and MNAR
37	Wang et al. (2023) *Stat Biopharm Res*	Missingness in mixed variables
39	Heussen et al. (2023) *Stat Biopharm Res*	Imputation that preserves randomisation
**Theme: Modelling and covariate adjustment approaches**		
47	Liu et al. (2020) *Stat Biopharm Res*	Model averaging approach
49	Moore et al. (2020) *BMC Med Res Method*	Bayesian methods
51	Zhou et al. (2020) *J Comput Graph Stat*	Bayesian procedure which account auxiliary covariates
53	Roochi and Mahabadi (2023) *Int JBiostat*	Bayesian methods for non-ignorable missingness
54	Liu and Yau (2021) *J Stat Plan Inf*	Autoregressive model linked with missing data mechanism
55	Li et al. (2022) *Contemp Clin Trials*	GLMM under MAR assumption
56	Ma et al. (2022) *Pharm Stat*	MI on continuous multivariate outcomes
58	Touraine et al. (2023) *BMC Med Res Method*	LMM and JM
60	Lin and Wang (2023) *Stat Methods Med Res*	Censored observation with MNAR assumption
62	Lin and Wang (2022)* Biom J*	Censored and non-ignorable missingness
66	Staudt et al. (2022) BMC Med Res Method	Latent growth modelling for multivariate longitudinal outcomes
68	Lu and Chen (2022) *Stat Methods Med Res*	Correlated outcomes in five different Markov chain Monte Carlo sampling algorithms
71	Van Lancker et al (2020) *Pharm Stat*	Binary primary short-term outcomes with covariates
75	Morris et al (2022) *Trials*	Prognostic covariance to increase precision
76	Wang et al (2023) *J Am Stat Assoc*	Model-robust estimator for different types of outcomes
**Theme: Simulation methods**		
79	Tang et al. (2022) *Contemp Clin Trials*	Progression-free survival in the presence of missingness
**Theme: Estimand methods**		
81	Liu et al. (2021) *Stat Biopharm Res*	Hypothetical estimands on Bayesian sensitivity analysis
82	Keene et al. (2021) *Contemp Clin Trials*	Limitations of ITT analysis
83	Van Lancker et al. (2023) *Stat Biopharm Res*	Hypothetical strategies
84	Jamoul et al. (2022) *BMC Med Res Method*	Impact of shutdown during pandemic in phase III cancer trials
86	Qu et al. (2021) *Pharm Stat*	Tripartite estimands under causal inference framework
87	Olarte Parra et al. (2023) *Stat Biopharm Res*	Causal effect estimator and missing data estimators
89	Kang et al. (2022) *Clin Trials*	Guidance of monitoring a trial

RBI: reference-based imputation; RTB: return to baseline; JR: jump to reference; MI: multiple imputation; MNAR: missing not at random; MAR: missing at random; PRO: patient reported outcomes; GLMM: generalised linear mixed model; LMM: linear mixed model; JM: joint model; ITT: intention to treat.

#### Missing value imputation methods

One approach that can be used when a trial is interrupted is to consider the data that would have been observed, had the trial been completed as missing and use missing value imputation methods. A number of imputation methods that could be applied in this setting, including the hot deck method, mean imputation and regression-based imputation, have been proposed (see, e.g. the books by Little and Rubin^8^ and Carpenter and Kenward^9^ and review paper by Carpenter and Smuk^10^), with multiple imputation (MI) approaches^11^ particularly common in practise in clinical studies.

A total of 20 of the 43 papers identified in our review focus on methods that involve missing data imputation. These are described below, giving a snapshot of recent advances in this area.

Statistical methods for imputation of missing data proposed in these papers are mostly parametric model-based. Deviation from the distributional assumptions made regarding observed and missing data can thus lead to biased inference. Fan et al^12^^*^ develop methods for mean rank imputation^13^ to handle missing data in non-normal outcomes and covariates in a two-arm longitudinal clinical trial that do not require distributional assumptions.

In handling longitudinal trials with missing data, the reference-based imputation (RBI) method is an important tool for sensitivity analysis. This method uses MI for the treatment group based on the model developed for the reference group.^14–16^ Lu^17^^*^ proposes an RBI procedure to relax the assumption from missing at random (MAR) to missing not at random (MNAR) in a longitudinal two-arm study. The method is developed by blending the variance estimation from the delta-adjusted pattern mixture model with delta adjustments fixed at the maximum likelihood estimate (MLE) to provide an effective estimation of the treatment effect. Bartlett^18^^*^ also considers an RBI approach, in this case, to estimate treatment effect in repeated measured randomised controlled trials (RCTs), comparing this with Rubin’s variance and repeated sampling variance. He favours RBI variance in a frequentist paradigm. Jin and Fang^19^^*^ propose RBI procedures to handle repeated incomplete binary outcomes using multivariate probit (MVP) and logistic models. The distribution of missing binary outcomes is explored and implemented. This paper gives a general framework to deal with repeated incomplete binary outcomes through RBI. Diao et al.^20^^*^ developed another reference-based MI procedure for modelling recurrent event data with informative dropout. In this work, two approaches have been considered for MI; the copy reference, based on the assumption that patients after discontinuation in both treatment and control arms have the same conditional distribution based on the completers in the control arm and jump to reference (JR), where, following dropout, the treatment mean is assumed to be equal to the control mean. A semi-parametric procedure is considered to model data and non-parametric MLEs are used to estimate the model parameters. Wolbers et al.^21^^*^ introduce a four-step conditional mean RBI procedure to deal with continuous longitudinal normal outcomes in a complex MAR set-up. Simulation is performed using Jackknife resampling techniques. This approach differs from conventional MI methods based on the Bayesian approach combined with Rubin’s rules.^22,23^ Garcia-Hernandez et al.^24^^*^ propose a framework for implementing RBI, JR and tipping-point analysis to longitudinal trials interrupted by intercurrent events (ICEs) using a treatment policy strategy. To do this they characterise the intervention discontinuation effect which quantifies the difference between the outcome of interest if ICE would not occur and the outcome regardless of drug discontinuation after dropout. Rabe and Bell^25^^*^ used simulation studies to compare methods of MIs, a linear mixed model (LMM), last observation carried forward imputation, and complete case analysis in terms of type I error rate, bias, and power for non-inferiority trials with longitudinal outcomes having missingness in intention to treat (ITT) and per-protocol (PP) populations.^26^ Data were simulated under different varying factors (e.g. outcome trajectory over time, proportion of missing data, compliance scenario, etc.) and analysed accordingly. Wang et al.^27^^*^ use simulation studies to assess five different methods of MI to handle missingness for continuous outcomes under MNAR assumptions after the occurrence of ICEs under the treatment policy strategy framework in which subjects are followed and accessed regardless of treatment compliance. They have also considered three case studies to show how three methods out of the five have an advantage in regulatory decision-making. Liu et al.^28^^*^ propose a distributional imputation procedure as a tool for sensitivity analysis to impute each missing observation using a target imputation model given the observed data for longitudinal outcomes with monotone missingness. This method is efficient theoretically and gives a consistent variance estimator.

In settings in which treatment effects are transient such as certain drug trials, the return to baseline (RTB) imputation method, in which unobserved data for both treatment and control groups are imputed using a model based on baseline observation, has been proposed owing to the hypothetical estimand which assumes that patients lose treatment benefit of treatment and RTB state when they dropout of the study. This motivates Jin^29^^*^ to propose a hybrid strategy for RTB to handle monotone missingness under both MAR and MNAR assumptions simultaneously. The analytic likelihood-based estimate is derived for a hybrid RTB approach to enhance the efficiency.

Currently used RTB imputation procedures inflate variance and are biased for estimating treatment effect when missingness depends on observed baseline and/or early outcomes. Qu and Dai^30^^*^ propose a new RTB procedure which can easily be implemented using existing statistical analysis packages in R and SAS. Simulation studies show that the new method is better than the existing methods in terms of bias and variance minimisation. Zhang et al.^31^^*^ describe statistical properties and find out the conditions of RTB analysis for longitudinal clinical trials under the ‘wash-out’ assumption that there will be no treatment effect once a patient drops out. The performance of the treatment effect under RTB is compared for different sample sizes (based on the RTB method and normal) and missing data mechanisms (missing completely at random (MCAR) and MAR). Jin and Liu^32^^*^ propose a two-level RTB method to impute missing daily patient-reported outcomes for longitudinal studies with monotone missingness. This method improves efficiency over the standard MI approach with Rubin’s rules.

Jin et al.^33^^*^ consider imputation of missing data in a longitudinal study with data recorded on a daily basis and assigned to ‘cycles’ of weeks or months, as seen in the studies of conditions such as migraine, chronic insomnia or menstrual cycles. Jin et al.^*^ developed a new two-level modelling approach to overcome the possible bias of the standard approach that arises due to cycle averages being treated as missing if the number of daily outcomes in the cycle is less than a prespecified limit.

MI methods were also developed by Yang et al.^34^^*^ in the specific setting of censored survival data. They constructed a general framework for sensitivity analysis relaxing the missing assumption mechanism and ensuring consistency of the variance estimator of the treatment difference. This work reduces the computational labour of existing MI procedures. Additional MI-based methods were developed by Jin et al.,^35^^*^ who propose a procedure for deriving the analytical likelihood-based approach under a combined hypothetical strategy for sensitivity analysis to handle missingness due to COVID-19 and non-COVID reasons to differentiate between MAR and MNAR mechanisms in monotone missingness. Another work by Chen et al.^36^^*^ on MI comes from the area of non-responder imputation (NRI), where non-response is treated as treatment failure in a binary outcome for some diseases. They propose a hybrid imputation approach combining NRI and MI to assess the impact of dropouts for different reasons (MAR and MNAR). The proposed procedure is more efficient than standard NRI and is also applicable to different data types including continuous, binary, and survival data. Missingness in a monotone fashion is often encountered in longitudinal studies with mixed variables (binary, continuous, time-to-event (TTE) and time-to-recurrent events). Wang et al.^37^^*^ propose an imputation procedure for this type of problem which is based on fully conditional specification.^38^ Huessen et al.^39^^*^ propose imputation methods that preserve randomisation so that randomisation-based inference methods can be used, for example, as in Edgington and Onghena.^40^

#### Modelling and covariate adjustment approaches

Statistical modelling is a useful technique for handling missing data including data missing due to the interruption of a trial as described above. A number of models have been proposed for the analysis of longitudinal data of different types, and are valid under a range of assumed missingness mechanisms. Examples are the mixed model for repeated measures^41^ in an RCT under MAR assumption for longitudinal continuous outcomes when the outcome vector is distributed as multivariate (MV) normal, generalised linear mixed models (GLMMs)^42,43^ and generalised estimating equation (GEE)^44^ models to deal with missingness of longitudinal binary outcomes.

Our review identified a number of articles reporting new modelling approaches. These were in specific settings or under specific assumptions, such as dropouts performing worse than observed or non-ignorable missingness beyond Gaussian outcomes, as described below.

On the assumption that dropouts perform worse than the observed outcomes for continuous outcomes in an RCT, usually the trimmed mean^45^ and median regression^46^ approaches are used. One of the significant drawbacks of these methods is that missing outcomes are ignored. Liu et al.^47^^*^ show how the overall treatment effect can be estimated using a model averaging approach,^48^ averaging effect estimates from two likelihood-based methods that use either a single normal or a mixture of two normals. Their approach is also shown to allow mild deviation from the normality assumption, but uses only baseline and final outcome rather than the full longitudinal outcome. Moore et al.^49^^*^ propose a Bayesian natural cubic B-spline varying coefficient method to account for non-ignorable dropouts in longitudinal studies for distributions of the exponential family form. Flexibility over the distribution of dropout times is enabled through Bayesian bootstrapping^50^ and the functional form of the relationship between regression coefficient and dropout time is modelled using natural cubic B-splines. The method is shown to improve the precision of the estimates in terms of mean squared error compared to other existing methods, which include selection, frailty and mixed models under some rigorous parametric assumptions.

Zhou et al.^51^^*^ developed a semi-parametric Bayesian procedure to handle monotone missingness for accounting auxiliary covariates. They factored the whole data set into two parts based on observable and missing values conditional on the mechanism of missingness (MAR/MNAR) and auxiliary covariates^52^ using extrapolation factorisation. Their proposed procedure incorporating covariates improves the robustness of the inference and reduces the extent of sensitivity analysis. It also gains precision over restrictive parametric and Bayesian non-parametric procedures. Roochi and Mahabadi^53^^*^ developed a Bayesian sensitivity analysis for longitudinal outcomes to non-ignorable missingness.

Liu and Yau^54^^*^ consider the problem of dealing with missing longitudinal data. Modelling outcomes at a given time as being dependent on the previous observation and covariates through an outcome probability function which is linked with a missing data mechanism, they propose the use of a first-order autoregressive (AR(1)) model to deal with the problem of non-ignorable missingness. They give expressions to enable model parameters to be estimated using the observed likelihood function and present a simulation study showing that the proposed method performs better than the naive method which ignores the non-ignorable missing mechanism.

Li et al.^55^^*^ compare the performance of GLMM and semi-parametric GEE approaches to deal with longitudinal binary outcomes with missingness under the MAR assumption, based on literature review and simulation studies. They also provide a set of recommendations for methods in this setting, including a two-step approach combining MI and GEE (MI–GEE) relevant when the binary outcome is obtained from dichotomisation of a continuous outcome and a high proportion of missingness and/or unbalanced missingness in two arms. Two common approaches to analyse data for incomplete binary outcomes in longitudinal clinical trials, dichotomised from continuous variables, are GLMM and MI-based methods. MI-based methods impute the missing continuous outcomes and then analyse the data through generalised linear model (GLM) after dichotomising at a threshold value. Based on simulation studies, Ma et al.^56^^*^ show that such MI-based methods outperform GLMM in terms of variance minimisation, when data are generated from continuous MV distributions including the MV normal, MV t , and log-normal distributions. The linear mixed model (LMM) is unable to produce unbiased estimates when informative dropout occurs in a longitudinal trial. A joint model (JM) involving dropout with longitudinal outcomes could effectively minimise this bias.^57^ Touraine et al.^58^^*^ compare LMM and JM methods using data obtained from a randomised phase II–III cancer clinical trial and show that LMM overestimates the effect parameter of interest in both arms (treatment and control), which can be avoided by considering a JM that allows the association between longitudinal outcome and dropout. Multivariate non-linear mixed model (MNLMM)^59^ methods are used to analyse multiple longitudinal outcomes which have non-linear trajectory patterns. However, this model fails to capture the presence of censored and non-ignorable missing outcomes. Lin and Wang^60^^*^ propose a new model MNLMM-CM which accounts for censored and MNAR simultaneously under the assumption of a non-linear trajectory of the outcome variable by exploiting a selection model^61^ and Taylor series expansion.

Lin and Wang^62^^*^ develop a method to analyse MV longitudinal outcomes subject to both censored and non-ignorable missing values when data may be MNAR. The selection model^63^ and logistic regression approaches are used to deal with this problem. Monte Carlo expectation conditional maximisation^64,65^ is adopted to compute MLEs.

Staudt et al.^66^^*^ demonstrate how to handle missing data under MAR and MNAR through sensitivity analysis in a randomised clinical trial using latent growth modelling.^67^

The multivariate probit (MVP) model is capable of handling correlated outcomes in longitudinal binary outcomes and imputing non-ignorable dropouts, which are very often encountered in clinical trials. Lu and Chen^68^^*^ compare the performance of five different Markov chain Monte Carlo sampling algorithms to exploit the correlation among the successive outcomes via simulation to estimate the treatment effect based on different criteria such as computational cost, robustness, and accuracy. They conclude that the Gibbs sampling algorithm^69^ and partial autocorrelation parameterisation approach^70^ are more efficient and flexible in this setting.

Covariate adjustment allows incorporation of and adjustment for prognostic covariates measured before randomisation in the analysis. This improves the robustness of inference on treatment effects. Van Lancker et al.^71^^*^ consider the setting of a trial with a binary primary outcome with baseline covariates and multiple short-term outcomes with the number of patients with later data observed diminishing over a series of interim analyses. Building on the methods of averaging the estimates obtained from different cohorts, they show how to improve the treatment effect estimator by utilising the covariate and short-term outcome data. Assuming that any association between the primary and either the short-term outcomes or the baseline data remains constant over time, using a test statistic obtained from a GLM, they calculate conditional power based on a Brownian motion structure.^72–74^ This enables futility stopping or sample size re-assessment without type I error rate inflation.

Morris et al.^75^^*^ compare the three approaches (direct adjustment, standardisation, and inverse-probability-treatment-weighting) to handle prognostic covariates to increase the precision of the treatment effect. They conclude that all methods deal with missingness in covariates with each method having advantages and disadvantages, and that the input of clinicians is very important in choosing the most appropriate method for a particular trial.

Wang et al.^76^^*^ showed that the robustness of power and precision of a trial can be enhanced by incorporating the initial condition of the disease or potential biomarker, which are correlated with the primary outcome of a trial. They combined two methods, namely, stratified randomisation^77^ and biased coin randomisation^78^ to find the estimator of treatment effect. Their work covers continuous, binary and TTE outcomes when some observations are MAR.

#### Simulation methods

Simulation methods are often used for assessment, validation or comparison of newly proposed approaches, including in some of the papers described above. In settings where modelling or imputation methods are hard to implement because of complex scenarios or assumptions, however, simulation techniques can provide an alternative to imputation approaches as described above.

Cancer patients are one patient group that needs regular hospital visits for tumour assessment and treatment administration. This was greatly affected during the SARS-CoV-2 pandemic, impacting ongoing oncology trials. Tang et al.^79^^*^ evaluated the impact of COVID-19 in such trials through simulations and prescribed remedial measures to take further decisions regarding design, data collection, and analyses. They also recommended a decision tree to choose a suitable method for progression-free survival (PFS) evaluation in the presence of missingness.

#### Estimand methods

Following the publication of the ICH E9(R1) framework,^80^ there has been increasing prominence in formalising the specification of estimands prior to the analysis of clinical trials, particularly in order to deal with ICEs. Our review identified a number of papers where estimands were discussed in the context of interruptions to clinical trials, as these form the basis for the statistical analysis for interrupted trials they are discussed here.

Liu et al.^81^^*^ consider two hypothetical estimands and explore Bayesian approaches to find point and interval estimators for the treatment effect of interest. Assuming a proportion of missing data follows a Dirichlet prior distribution, they propose Bayesian sensitivity analysis and compare this approach with the existing likelihood-based and MI methods.

Keene et al.^82^^*^ discuss the limitations of ITT analysis in the light of the estimand framework in the specific context of clinical trials affected by the SARS-CoV-2 pandemic. They also describe alternative analysis protocols, for example, PP analysis, of which a variant is the ‘as treated’ (AT) analysis.^26^ Despite having the guidelines^80^ in framing estimands to deal with ICEs, there are some unforeseen ICEs which disrupt the procedure of conducting and accruing data of a trial as discussed.^2–5^ Van Lancker et al.^83^^*^ introduce three relevant hypothetical strategies pertaining to SARS-CoV-2 pandemic-related ICEs and review the treatment effect estimates in a range of scenarios based on causal inference and missing data methods. They also describe the framework for the analysis of future trials that might face similar interruptions. Jamoul et al.^84^^*^ provide simulation studies to understand the impact of a shutdown during the pandemic in phase III cancer trials using PFS. They consider two different strategies, namely ‘treatment policy’ and ‘hypothetical’ and conclude that the former conforms with the ITT principle and should be recommended for minimising bias and increasing power.

Defining an appropriate estimand for the treatment effect in a clinical trial is crucial, especially for trials affected by ICEs. A tripartite estimand^85^ takes into account the benefit of all stakeholders (patients, prescribers, payers, sponsors and regulators) of a trial. In a paper identified in this review, Qu et al.^86^^*^ discuss ways to estimate and interpret tripartite estimands under a causal inference framework, illustrating implementation of the method through a phase III clinical trial in type 1 diabetes. Olarte Parra et al.^87^^*^ consider a hypothetical strategy where the treatment effect assumes that ICEs are somehow prevented from occurring. Based on this, they explore causal inference (see Ding and Li^88^ and references therein) and missing data methods for treatment effect estimation and establish the link where in some situations causal effect estimators become identical with missing data estimators. Kang et al.^89^^*^ show how the choice of an appropriate estimand is aligned with the statistical analysis plan, providing general systematic guidance for monitoring a trial.

## 4. Discussion

Although interruptions can occur for many reasons, a consequence of the impact of the particular challenges of the SARS-CoV-2 pandemic has been a considerable increase in the number of clinical trials that have been interrupted. Many of these trials now have incomplete data sets, presenting a challenge in their analysis. This paper reports a review of the methodology available to address this challenge. The review has focused on relevant methodological work published in 21 statistical methodology journals in the years 2020 to 2023 and thus identifies methods motivated by the SARS-CoV-2 pandemic as well as provides a snapshot of recent relevant work.

The review identified 43 papers. These are briefly summarised in Table 1 and marked with an asterisk both in the text and in the reference list. These were categorised into four broad themes of *Missing value imputation methods*, *Modelling and covariate adjustment approaches*, *Simulation methods* and *Estimand methods*. Although these themes are, to some extent, arbitrary, and the work could have been categorised in different ways, we feel that this categorisation is helpful in specifying key methods being developed and applied for use in interrupted clinical trials, and also gives an indication of how specific methods described in the papers identified in the review could be generalised or applied in a broader context. It has also enabled us to identify important references on which the newer work reported in detail here is based.

In some cases, the categorisation of articles identified in the review to one of our chosen themes was not unique. For example, the procedure proposed by Lu^17^^*^ provides an effective treatment effect estimand, but as the primary objective of the work to give a variance estimate is based on an imputation procedure, this paper has been included under the *Missing value imputation methods* theme. The split of papers across the four categories is roughly equal between missing value and modelling methods. This is probably not unexpected and perhaps reflects how applied statisticians might naturally approach the problem of an interrupted trial, whether pre or post pandemic. We found only one paper that we could assign to the *Simulation methods* category. We assigned a paper to this category only if the method proposed was based on the use of simulations. Many authors used simulations to assess methods, but these were categorised according to the type of method proposed and assessed, such as MI or modelling. The *Estimand methods* theme is rather different to the others in that papers do not necessarily focus solely on proposing particular statistical methods, but rather on how the treatment effect should be interpreted when a trial is interrupted.

The nature of interruption will be the key factor for deciding the methodology to be considered. Table 1 above could be helpful in identifying methods appropriate in a particular setting.

Although our focus has been on interruptions caused by the SARS-CoV-2 pandemic, and the time window used in our review means that this is also the context of most of the work included, many of the approaches proposed or evaluated could be used, possibly with some extension, in settings with interruptions arising from other causes, as is made explicit in some of the papers reviewed that deal specifically with interruptions due to COVID and non-COVID cases. It is worth mentioning the Ingram Olkin forum sessions organised by the National Institute of Statistical Sciences^90^ to explore methods to deal with unplanned clinical trial disruptions, focusing specifically on the areas of estimands and estimation (Van Lancker et al.^83^), use of auxiliary source of data (Calderazzo et al.^91^), randomisation tests (Uschner et al.^92^), and Bayesian and frequentist approaches (Kunz et al.^93^). This work was motivated by the SARS-CoV-2 pandemic, but the objective was to deal also with trials disrupted for other non-pandemic-related reasons.

Missingness of data due to the SARS-CoV-2 pandemic have usually been considered as MAR or MCAR, but in other settings, MNAR could be relevant depending on the disease area under consideration and the data accrual process. In general, different causes of interruptions could lead to different types of missingness, and hence to different statistical approaches. This could thus be a motivation for further methodological work and could lead to a taxonomy of methods for handling interruptions of different types arising from different causes.

The methods considered so far could also be extended in terms of the distributional assumptions made regarding missing and partially observed data and/or covariates. A different approach could be to use a surrogate or secondary outcome in place of the primary outcome if this is available for a greater number of patients in the trial, though in this case, it is important to assess carefully whether this will address the research question under consideration, along with ensuring that bias and precision are controlled. One example of this approach is the trial reported by Metcalfe et al.^94^ In this case, an outcome that required post-surgery clinical assessments was replaced by a secondary outcome that could be obtained remotely without requiring a hospital visit when COVID-19 restrictions made this impossible.

In some clinical trials, even if data are observed, the effect of COVID-19 can lead to uncertainty regarding the reliability of those data. Examples are the AFFIRM-AHF^95^ and IRONMAN^96^ studies. In such cases, alternative approaches including sensitivity analyses can be considered.^97^

## Appendix 1. Scopus search terms used in literature search

The literature search used the following search term to identify relevant methodological articles within the specified period.

TITLE-ABS-KEY (clinical AND trials) AND TITLE-ABS-KEY (dropout OR missing OR interrupt) AND SRCTITLE ((australian AND new AND zealand AND journals AND of AND statistics) OR (bmc AND medical AND research AND methodology) OR (biometrical AND journal) OR (biometrics) OR (biometrika) OR (biostatistics) OR (canadian AND journal AND of AND statistics) OR (clinical AND trials) OR (computational AND statistics AND data AND analysis) OR (contemporary AND clinical AND trials) OR (journal AND of AND the AND american AND statistical AND association) OR (journal AND of AND applied AND statistics) OR (journal AND of AND biopharmaceutical AND statistics) OR (journal AND of AND causal AND inference) OR (journal AND of AND computational AND graphical AND statistics) OR (journal AND of AND the AND royal AND statistical AND society AND series AND a) OR (journal AND of AND the AND royal AND statistical AND society AND series AND b) OR (journal AND of AND the AND royal AND statistical AND society AND series AND c) OR (journal AND of AND statistical AND computation AND simulation) OR (journal AND of AND statistical AND planning AND inference) OR (pharmaceutical AND statistics) OR (scandinavian AND journal AND of AND statistics) OR (sequential AND analysis) OR (stata AND journal) OR (r AND journal) OR (statistics AND in AND biopharmaceutical AND research) OR (statistics AND in AND medicine) OR (statistical AND methods AND in AND medical AND research) OR (trials)) AND PUBYEAR > 2019 AND PUBYEAR < 2024 AND (LIMIT-TO (DOCTYPE, “ar”)) AND (LIMIT-TO (SUBJAREA, “MEDI”) OR LIMIT-TO (SUBJAREA, “MATH”) OR LIMIT-TO (SUBJAREA, “PHAR”)).
